# Group intervention for siblings and parents of children with chronic disorders (SIBS-RCT): study protocol for a randomized controlled trial

**DOI:** 10.1186/s13063-020-04781-6

**Published:** 2020-10-14

**Authors:** Krister W. Fjermestad, Wendy K. Silverman, Torun M. Vatne

**Affiliations:** 1 Department of Psychology, University of Oslo, New Haven, Norway; 2 Child Psychiatry, Child Study Center, New Haven, USA; 3 Department of Psychology, Yale University, New Haven, USA; 4 Frambu Resource Centre for Rare Disorders, Siggerud, Norway

**Keywords:** Randomized controlled trial, Study protocol, Group intervention, Siblings, Young carers, Neurodevelopmental disorders, Family communication, Mental health

## Abstract

**Background:**

Siblings and parents of children with neurodevelopmental disorders are at risk of mental health problems and poorer family communication. Some group interventions for siblings exist, but few have clearly described parent components and none are considered evidence-based.

**Methods:**

We are conducting a randomized controlled trial comparing a five-session manual-based group intervention for siblings (aged 8 to 16 years) and parents of children with neurodevelopmental disorders to a 12-week waitlist, called SIBS-RCT. The intervention comprises three separate sibling and parent group sessions and two joint sessions in which each sibling talks to their parent alone. The intervention aims at improving parent-child communication and covers themes such as siblings’ understanding of the neurodevelopmental disorder, siblings’ emotions, and perceived family challenges. Participants are recruited through municipal and specialist health centers across Norway. The primary outcome is sibling mental health. Quality of life and family communication are secondary outcomes. Participants are block-randomized to the intervention or 12-week waitlist in groups of six. Measures are collected electronically at pre- and post-intervention/waitlist, as well as 3, 6, and 12 months post-intervention. The main effect to be examined is the difference between the intervention and waitlist at 12 weeks post. All outcomes will also be examined using growth curve analyses. We plan to include 288 siblings and their parents by the end of 2022.

**Discussion:**

SIBS-RCT represents a major contribution to the research and practice field towards establishing an evidence-based intervention for siblings. In the event that intervention and waitlist are no different, the impact of SIBS-RCT is still substantial in that we will aim to identify participant subgroups that show positive response and effective components of the SIBS manual by examining group leader adherence as an outcome predictor. This will allow us to continue to re-engineer the SIBS manual iteratively to improve outcomes, and avoid the promotion of a less-than-optimal intervention.

**Trial registration:**

ClinicalTrials.gov NCT04056884*.* Registered in August 2019

## Administrative information

The order of the items has been modified to group similar items (see http://www.equator-network.org/reporting-guidelines/spirit-2013-statement-defining-standard-protocol-items-for-clinical-trials/).

**Table:** 

Title {1}	Group intervention for siblings and parents of children with chronic disorders (SIBS-RCT): Study protocol for a randomized controlled trial
Trial registration {2a and 2b}.	ClinicalTrials.gov. Project identifier: **NCT04056884**
Protocol version {3}	Version 1; September 2019
Funding {4}	Internal funding by the main project organizers: Department of Psychology, University of Oslo, Norway: and Frambu Resource Centre for rare disorders, Norway. In-kind contributions from intervention sites (i.e., no major external funding sponsor).
Author details {5a}	Department of Psychology, University of Oslo, Norway, and Frambu Resource Centre for rare disorders, Norway.
Name and contact information for the trial sponsor {5b}	Department of Psychology, University of Oslo, Po Box 1094 Blindern, 0317 Oslo, Norway. joakim.dyrnes@psykologi.uio.no Frambu Resource Centre for rare disorders, Sandbakkveien 18, 1404 Siggerud, Norway. krk@frambu.no National Advisory Unit for Rare Disorders, Oslo University Hospital, Kirkeveien 166, Oslo, Norway. sjeldne-diagnoser@ous-hf.no
Role of sponsor {5c}	The sponsors are jointly responsible for design, data collection, data analyses and results dissemination. All contributing intervention sites can use their own data, after agreement with the project management.

## Introduction

### Background and rationale {6a}

Sibling relationships, our longest lasting bonds, represent a unique source of learning, support, and rivalry. Chronic illness in children involves risk for reduced well-being among siblings [1]. The mechanisms behind this risk are associated with (1) the chronic illness itself (e.g., behavior problems), (2) siblings’ lack of illness knowledge (e.g., misunderstandings), (3) poor parental mental health due to extra care responsibilities, and (4) poor family communication (e.g., less open and warm communication [2–4]).

Previous sibling interventions have shown positive outcomes for siblings’ well-being (e.g., [5, 6]). However, systematic reviews have demonstrated that no intervention has an established evidence-base [7, 8]. Therefore, we plan to examine a group intervention for siblings and parents of children with chronic illness in a randomized controlled trial (SIBS-RCT). SIBS-RCT is based on preliminary studies conducted by our consortium. First, we conducted a qualitative study of group sessions for 58 siblings of children with rare disorders. We found that siblings have limited disorder knowledge [4], conflictual emotions and passive coping strategies [9], and unique perspectives on their brothers and sisters with disorders [10]. Based on user feedback and these preliminary findings, we developed the SIBS intervention, a 5-session manual-based program with parallel sibling and parent groups. A unique feature of the SIBS intervention is integrated sibling-parent dialogs, which we added due to evidence of poor family communication in families of children with chronic illness [2].

We examined the SIBS intervention in an open trial with 107 families of children with rare disorders, autism, congenital heart disease, cerebral palsy, or Down syndrome. We found significantly improved sibling mental health and family communication from pre-intervention to 6-month follow-up (mean effect size *d* = 0.43) [11]. Acceptability and feasibility were promising, with a 54% response rate, 86% group leader manual fidelity, and mean sibling and parent satisfaction scores of 3.6 on a 1–4 rating scale.

Examining the effects of the SIBS intervention in an RCT design, as proposed herein, is the next critical step to establish it as an evidence-based intervention for siblings. We focus on neurodevelopmental disorders (ND), defined by impaired development of the central nervous system, affecting behavior, physical abilities, and mental health [12]. We decided on ND for three main reasons. First, ND represent a large group of physical and mental health conditions (e.g., autism spectrum disorders, cerebral palsy, epilepsy, intellectual disability), which enables our results to be generalized across conditions. Second, evidence suggests the impact of chronic illness on siblings is transdiagnostic and not disorder-specific [7]. Therefore, including across the ND spectrum is reasonable in comparison with focusing on specific ND diagnoses only. Third, the impact of ND represents a major public health challenge, as up to 15% of children have ND, and many of these have at least one typically developing sibling [12]. We present important advances of the current SIBS-RCT relative to past trials in Table 1.

**Table 1:** Methodological advances of current SIBS trial relative to past sibling intervention trials

Documented limitations^a^	SIBS study features
Few controlled trials^b^	Randomized controlled design
Small sample sizes/insufficient power	A sample of 288 siblings based on power analysis
Unclear inclusion criteria	Child with ND enrolled in health services is the main inclusion criteria
Lack of standardized measures	Well-validated measures
Single raters only (most often parents)	Multi-informant (siblings, parents, teachers, clinicians, observers)
Cross-sectional/lack of follow-up data	Prospective with multiple assessment points up to 12 months post
No session description/protocol/manual	Detailed session-by-session manual
Unclear description of parent involvement	Parallel and joint parent component
No adherence data	Group leader adherence and competence measured

^a^Based on reviews ([7] 17 studies and [8] 16 studies)

^b^One randomized controlled trial has been conducted (*N* = 21) [5]

### Objectives {7}

Objective 1 is to promote sibling well-being by addressing research question 1: *Does the SIBS intervention improve mental health for siblings of children with ND compared to waitlist?* and research question 2: *What are the long-term trajectories of sibling well-being, in terms of mental health, family communication, quality of life, sibling adaptation, and disorder knowledge from pre-intervention to 12-month follow-up?* Based on the findings from our open trial, we hypothesize that the SIBS intervention will outperform waitlist and that the mean 12-month trajectories will indicate significantly improved well-being. Objective 2 is to identify those siblings who are most likely to benefit from the SIBS intervention by addressing research question 3: *Which factors (i.e., ND impairment, parent mental health, family stress) predict outcomes of the SIBS intervention?* Previous findings indicate higher ND impairment and poorer parent mental health will be associated with poorer sibling mental health [1, 3]. However, there is limited knowledge upon which to base hypotheses about predictors of effects. The third research question is therefore exploratory. SIBS-RCT represents a major contribution to the research and practice field towards establishing an evidence-based intervention for siblings. In the event that intervention and waitlist are no different, the impact of SIBS-RCT is still substantial in that we will aim to identify (a) participant subgroups that show positive response and (b) effective components of the SIBS manual, by examining group leader adherence as an outcome predictor. This will allow us to continue to re-engineer SIBS-RCT iteratively to improve outcomes, and avoid promotion of a less-than-optimal intervention.

### Trial design {8}

SIBS-RCT is a randomized controlled trial comparing a 5-session group intervention for siblings and parents of children with ND to a 12-week waitlist comparator control. We decided on a 12-week waitlist as this is the longest permitted wait for intervention for internalizing and externalizing problems in the Norwegian public mental health system. For this reason, and because some participants may have high clinical symptom levels, a longer waitlist would be unethical.

## Methods: participants, interventions, and outcomes

### Study setting {9}

SIBS-RCT is conducted across ten sites covering urban and rural areas in Norway (see Fig. 3). We include specialist and municipal health services as both tiers provide services to children with ND, and both are required by legislation to address siblings’ needs. All sites volunteer to SIBS-RCT. A full list of sites can be obtained by contacting the first author.

### Eligibility criteria {10}

The inclusion criteria are as follows: (1) being the sibling of a child diagnosed with ND who is aged 0 to 18 years and who receives specialist and/or municipal health services. The service-recipient criterion has been set to ensure ND impairment is in the clinical range, as ND impairment is a consistent predictor of sibling well-being [1]; (2) sibling age 8–16 years, to ensure generalizability across age spans. This age range was used in our open trial [11]. Sibling groups will be age-clustered to ensure a maximum 4-year age span from the youngest to the oldest participant; and (3) one parent able to attend the intervention. Families choose which parent. We applied this in our open trial due to the care needs of the child with disorder, with two thirds of participating parents being the mother (34% fathers). We collect data from both parents regardless of participation and encourage the participating parent to discuss the intervention with the non-participating parent. The follow-up questionnaire addresses the involvement of the non-participating parent, and we control for participating parent in all analyses of outcomes. If more than one sibling from the same family attends, both parents can participate.

The exclusion criteria for siblings are as follows: (1) being enrolled as primary patients in specialist health services and (2) ND diagnosis. Eligibility criteria for group leaders are as follows (1) employment in municipal or specialist health services, (2) trained as a health professional (e.g., nurse, social worker, special educator, psychologist); and (3) completion of a training package comprising a 5-session e-learning course (which takes about 1 h to complete) and a 2-day practical workshop. Throughout the data collection period, we invite all group leaders to supervision webinars 2–3 times per year. We also arrange regular site visits where practical matters and questions about the intervention are discussed.

### Who will take informed consent? {26a}

Potential participants are invited via letters, phone calls, and/or personal communication at each site. Invitation letters include contact information for the study coordinator, who sends a link to the electronic consent form to families who contact him. Participants receive written information about the trial via this link and consent by signing the consent form electronically, using their personal identifying number and electronic ID (e.g., bank cards). Informed consent from one parent is required for children 8–16 years. Children above 16 years provide separate consent.

### Additional consent provisions for collection and use of participant data and biological specimens {26b}

No biological data are collected. For participants with a sibling with ND who is aged 16 and above, the sibling with ND has to consent for use of information about him/her (but not to family members joining the trial).

### Interventions

#### Explanation for the choice of comparators {6b}

Upon careful consideration of various potential comparator arms—a thorny issue in RCT research—we decided on a waitlist control design for three main reasons. First, waitlist represents a “treatment as usual” control comparator and provides an opportunity to demonstrate what the SIBS intervention adds to usual care. Second, empirical data that show how siblings’ well-being develops over time without intervention are lacking. Given that no care is the common scenario, a waitlist comparator is particularly reasonable at this stage of knowledge development. Third, it would be unethical to offer a comparator control intervention without robust preliminary outcomes, such as the SIBS intervention.

#### Intervention description {11a}

**Fig. 1:**
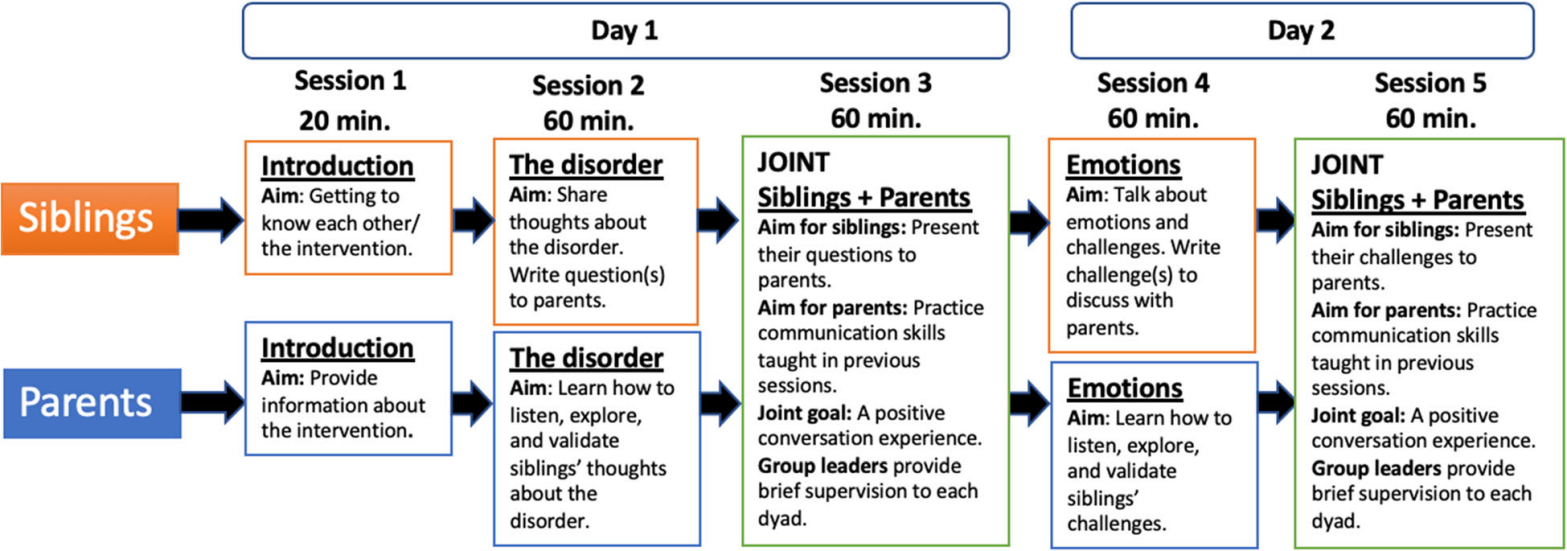
Overview of the SIBS intervention

The intervention comprises 5 sessions delivered over 2 days, 1 week apart. Day 1 comprises sessions 1–3 (3.0 h including breaks). Day 2 comprises sessions 4–5 (2.5 h including breaks). Sessions 1, 2, and 4 are parallel (separate) group sessions for siblings and parents. Sessions 3 and 5 are integrated sibling-parent dialogs in which each sibling and parent talk together, separate from other participants. Two main themes, both empirically derived sibling challenges, provide the basic components of the intervention: disorder knowledge and emotional experiences. These themes are discussed in relation to the overarching component, family communication. Session 1 is the introduction. Sessions 2–3 focus on siblings’ ND knowledge. Sessions 4–5 focus on siblings’ emotional experiences based on cognitive-behavioral principles about how thoughts and behaviors influence emotions. In sessions 2 and 4, siblings generate lists of ND questions and challenges, respectively, which a group leader then brings to the parent group for discussion. In sessions 3 and 5, each sibling discusses their questions and challenges with their parent. The parent sessions focus on communication training focused on ND knowledge and siblings’ emotional challenges, introducing the SIBS motto “listen, explore, validate,” using standardized video examples of real sibling-parent dialogs with various degrees of open-exploratory communication as a basis for discussion. Parent sessions also comprise psychoeducation about sibling challenges and introduction to cognitive-behavioral principles about emotional coping. The SIBS intervention is outlined in a step-by-step manual for group leaders (see Fig. 1).

Each group is conducted by two group leaders. Thus, running the intervention requires four group leaders. In sessions 3 and 5, one group leader provides feedback to each of the sibling-parent dialogs, reinforcing open-exploratory communication. All sessions are videotaped. We measure group leader adherence with the Competence and Adherence Scale for Cognitive Behavioral Therapy (CAS-CBT) [13].

#### Criteria for discontinuing or modifying allocated interventions {11b}

Because the intervention is conducted over two half days, there are no explicit criteria for discontinuing or modifying the intervention. If a family does not show up on the second course day, or do not submit questionnaires, we attempt to continue to collect data from these participants to examine trajectories of change based on partial data.

#### Strategies to improve adherence to interventions {11c}

We measure group leaders’ adherence to the manual with an adapted version of CAS-CBT. We take the following measures to enhance group leader adherence: quality training of group leaders. The training of group leaders comprises a 1h e-learning program, which the group leaders can access from anywhere online. The e-learning program was developed in close collaboration with users, i.e., parents of children with disorders and health care providers. After completion of the e-learning program, potential group leaders submit a reflection note about the SIBS manual content. When the e-learning course is approved, group leaders are invited to a 2-day practical workshop. So far, we have been able to provide this training free-of-charge for participants. The 2-day training course comprises practical run through of all the group sessions, with practical role plays after watching example videos from the manual developers. Throughout the study period, we conduct 2–3 webinars per year, which all group leaders are invited to. The purpose of the webinars is to present and discuss potential challenging issues in group delivery.

In terms of participant adherence, the SIBS manual includes a task in which parents and siblings sign a contract specifying a date and time for when they will continue the dialog started during the intervention. At the 12-month follow-up, we ask participants to self-report whether this happened or not. We use this measure as a proxy for participant adherence in our analyses of long-term outcomes. We also ask participants to complete a “quiz” about the main components of the SIBS manual.

#### Relevant concomitant care permitted or prohibited during the trial {11d}

Given the inclusion key is being siblings/parents of children with ND who receive specialist or municipal health services, concomitant care is unavoidable and disallowing it would be unethical. We register concomitant care for siblings at all measurement points. The intent-to-treat sample will be compared with the per-protocol analyses excluding participants who have received concomitant care.

#### Provisions for post-trial care {30}

If clinical needs beyond the SIBS intervention are identified among participants (e.g., suicidality), site coordinators will assist families with adequate referrals. Patient rights according to specialist and municipal health legislation applies in terms of participant risks.

### Outcomes {12}

The primary outcome is sibling mental health, measured with the Strengths and Difficulties Questionnaire (SDQ) [14]. The 25-item SDQ is rated by siblings (self-report), as well as the participating sibling’s parents and main teacher, as proxy reports on sibling well-being. The SDQ covers five domains (emotional, conduct, inattention, peer problems, and prosocial behavior). We chose the SDQ as the primary outcome for the three main reasons. First, the SDQ has well-documented psychometric properties with this age group and substantial overlap with lengthier mental health measures (e.g., [15]). In our open trial, the SDQ showed good inter-item reliabilities (*α* = .77 to .82) and significant reduction from pre-intervention to 6-month follow-up, demonstrating good responsiveness to change. Second, the SDQ provides a continuous score and a clinical cutoff score. This enables us to report both mean change scores from pre-intervention to post-intervention, 3-, 6-, and 12-month follow-up, and the number of participants in the clinical, borderline, and normal range at each measurement point. Third, the SDQ includes strengths, an essential component for measuring well-being. Outcomes will be reported for sibling, parent, and teacher ratings.

Secondary outcomes cover additional risk factors for siblings linked to the World Health Organization’s well-being definition [16]: (1) family communication, because poor family communication is a documented risk factor in families of children with chronic illness [2]. It is measured with the Parent-Child Communication Scale (PCCS) [17], rated by siblings about parents and parents about siblings; (2) quality of life, because it represents a function-based indicator of well-being beyond mental health symptoms. It is measured with the KINDL Fragebogen [18] rated by siblings and parents (about siblings); (3) sibling adaptation, because it is a well-being variable tailored for siblings of children with chronic illness. It is measured with the Negative Adjustment Scale (NAS) [19], rated by siblings; and (4) sibling ND knowledge, because it is associated with sibling well-being [4]. It is measured with the Sibling Knowledge Interview (SKI) [6], conducted with siblings over the phone by trained clinicians. All secondary outcomes have demonstrated internal consistency (*α* = .90 for KINDL [18]; *α* = .75 to .82 for PCCS [11, 17]; *α* = .79 for NAS [6]; *α* = .78 for SKI [11]).

Predictor variables are as follows: (1) ND characteristics, because ND impairment is a consistent predictor of sibling well-being [1]. Parents report about the child with ND with the well-validated Developmental Behaviour Checklist [20]. We have ethical approval to collect patient record information for the child with ND, from which we obtain a ND impairment score (i.e., diagnosis, clinician’s severity ratings, and number of clinic appointments); (2) parent mental health, because it is a documented predictor of sibling well-being [3]. It is measured with the well-documented Symptom Checklist-90 [21] and with the Beck Depression Inventory, for a more specific depression measure [22]; (3) family stress, due to the documented strain for families of children with ND. It is measured with the Family Support Scale [23]; and (4) group leader manual adherence, because a limitation of previous sibling intervention studies is the lack of adherence data, and to identify manual components that may need to be added/enhanced after the project period. We measure it using an adapted version of the CAS-CBT [13] (see Table 2).

**Table 2:**
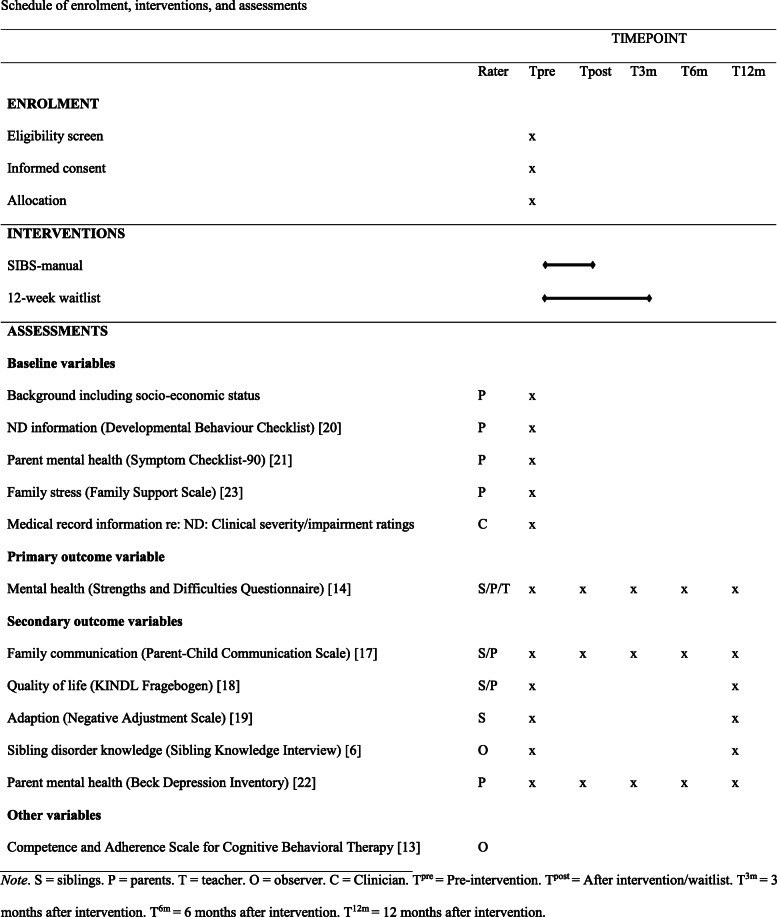
Schedule of enrollment, interventions, and assessments

*S* siblings, *P* parents, *T* teacher, *O* observer, *C* clinician, *T*^*pre*^ pre-intervention, *T*^*post*^ after intervention/waitlist, *T*^*3m*^ 3 months after intervention, *T*^*6m*^ 6 months after intervention, *T*^*12m*^ 12 months after intervention

### Participant timeline {13}

**Fig. 2:**
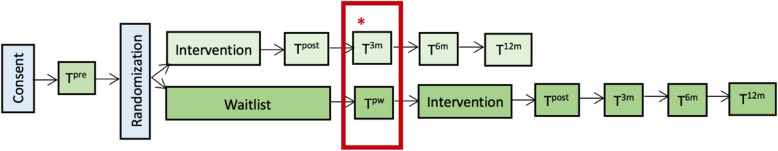
Participant flow chart. T^pre^, pre-intervention; T^post^, immediate post-intervention. T^pw^, post-waitlist; T^3m^, 3 months post; T^6m^, 6 monhts post; T^12m^, 12 month post. *The main group difference to be examined here

Siblings and parents of children diagnosed with ND who receive municipal or specialist health services are invited. The intervention is delivered at municipal and specialist health service sites. The intervention is the only activity requiring site attendance (two visits). All sibling- and parent-rated measures are completed online (see Fig. 2 for participant timeline; see Table 2 for the overview of the measures, measurement points, and alignment with study objectives).

### Sample size {14}

The planned sample is 288 siblings, 288 mothers, and 288 fathers. Based on a power analysis of change in the main outcome variable (SDQ) from our open trial, with a minimal relevant standardized effect size of 0.4, a statistical power of .8, and a significance level of .05 [24], we need a sample of 136 families. The intra-cluster correlation coefficient (ICC) in our open trial was 0.11. We have taken this lack of independence between observations into account in sample size estimates. The planned group size is 6, which results in an inflation factor (IF) of 1.55 (IF = 1 + (*m* − 1) × ICC) [25]. Adjusting with this IF resulted in a sample size of 211 (IF × 136). We further increased the sample to 288 to compensate for dropouts and concomitant care (estimated to 27% based on our open trial). An estimated 5000 children with ND aged 0–18 years receive health services in the catchment area, of whom an estimated 1000 (20%) have at least one sibling aged 8–16 years. Thus, a 29% response rate is required to reach our sample size. We believe this is a realistic goal given the response rate in our open trial was 58%, and whereas one site recruited 107 participants in our open trial, we now have eight sites.

### Recruitment {15}

Participants are recruited from municipal and specialist health services from August 2019 to December 2022. At each site, a coordinator will send invitations to all families of patients with ND aged 0–18 years registered in the patient database. During the study period, families of all referred patients who meet the inclusion criteria will be invited. Posters/leaflets with study information will be displayed at site waiting rooms. Recruitment will be monitored through continuous registration in a database and monthly meetings between the PI and site coordinators. The main feasibility challenge to SIBS-RCT is recruitment delay, which we will endeavor to allay by advertising broadly and involving multiple sites. Families with ND experience considerable strain and may struggle to attend interventions. We add the following features to enhance attendance: (a) only one parent required, (b) group session times advertised early (all sites have set intervention dates throughout the study period), and (c) attendance certificates to document siblings’ school absence. Group leaders are also under strain due to service requirements. We add the following features to enhance group leader recruitment/retention: (a) free-of-charge training and supervision, including online support; (b) formal contracts with sites; (c) regular site visits; (d) collaboration across sites; and (e) a trained team of advanced psychology students who can step in at sites free-of-charge. If delays are severe, we may add sites and advertise via newspapers (already ethically approved).

### Assignment of interventions: allocation

#### Sequence generation {16a}

Participants are randomly assigned to either intervention or 12-week waitlist (1:1), in clusters of 6, stratified by participant age to avoid > 4-year age gaps between participants in the intervention groups. Each site requests a randomization number when the site has ~ 6 consenting participants within each age range. A pre-generated randomization indicates whether the cluster will be either intervention or waitlist. The allocation sequence was computer-generated by a research coordinator who is independent of the PI. The sites and participants are advised of their allocation approximately 2 weeks prior to group dates, to respect their time management needs (e.g., planning time off school and work for participants; allowing time for other clients for group leaders). Participants allocated to waitlist receive the intervention after 12 weeks. The dates for the potential waitlist groups are included in the original invitation letter to ease families’ planning.

#### Concealment mechanism {16b}

The only person who knows the allocation sequence is the research coordinator, who keeps the sequencing on a password-locked computer and only reveals the allocation when the site has sufficient participants to run a group.

### Implementation {16c}

Eight sites are participating to date. The number of sites may increase depending on recruitment speed and funding resources available. Because the sites are geographically and organization-wise spread, the routines for enrollment are adjusted to the routines of the participating sites. The overall strategy is to include as many families as possible who fit the inclusion criteria. Because the sites are organized differently, this goal is reached in slightly different ways for different sites. Some sites send invitations to all families in their databases. Some sites send personal invitations to families known to them. Some sites advertise the trial with posters in waiting rooms. Some sites use a combination of these inclusion ways. Later in the project period, we may also consider advertising the study to patient-user associations and in local media (e.g., newspapers), and we have ethical approval to do so. The advantage of this flexible strategy is that recruitment is faster. The main disadvantages include that we will not be able to calculate an exact response rate, and the feasibility outcomes of the trial may be a bit tricky to interpret. That is, the extent to which our intervention fits “usual care” is more difficult to assess when our enrollment strategy is so flexible.

Across sites, the general procedure principle is that each site coordinator contacts the central project coordinator when they have at least 5 consenting participants. For each intervention group, 3–8 consenting families are required to allocate a randomization number. Participants receive all pre-intervention questionnaires approximately 1 month before the set group date, with regular reminders if not completed. Approximately 2 weeks before the set group date, families and group leaders are informed by the central research coordinator about whether the group was allocated to intervention or waitlist. If assigned to waitlist, participants receive the baseline questionnaires again 2 weeks before the intervention date (approximately 12 weeks after the initial group date). The aim is to conduct 48 groups over the project period. We have pre-generated a randomization list with 100 numbers, ensuring that there will never be more than 3 allocations to the same arm in a row (i.e., intervention or waitlist). This way, the number of participants in both arms will be somewhat balanced if we have to end the trial before reaching our aim of 48 groups, or if we need to increase the number of groups due to < 6 families per group (the aim is 288 families, i.e., 6 × 48).

### Assignment of interventions: blinding

#### Who will be blinded {17a}

Masking of participants, assessors, and group leaders is not possible as the control is waitlist and most measures involve self-report. Thus, emergency unmasking procedures are not applicable.

#### Procedure for unblinding if needed {17b}

This is not applicable since masking is not possible.

### Data collection and management

#### Plans for assessment and collection of outcomes {18a}

Primary and secondary outcomes are collected at pre- and post-intervention, plus 3-, 6-, and 12-month follow-up. Participants randomized to waitlist repeat outcome measures after waitlist (see Table 2 for details). While a 12-week waitlist weakens our ability to prove long-term effectiveness of the SIBS intervention, we find it unethical to make vulnerable siblings wait > 12 weeks for an intervention we expect will reduce their symptoms. All questionnaires are collected electronically using “Nettskjema,” an online questionnaire system run by the University of Oslo IT Department. Participants receive a link to the questionnaires via email. The system has built-in features to detect and prevent data entry errors and missing data. To help reduce/prevent dropout, several reminders are sent to participants at every measurement point. If a participant misses a measurement point, they still receive an invitation and reminders at the next measurement point (unless they actively withdraw from the study). Children can respond via their own or their parents’ email address.

#### Plans to promote participant retention and complete follow-up {18b}

Reminders are sent to all participants at each measurement point, via SMS and/or email, depending on participants’ preferences. To collect as much data as possible, a backup electronic version of only the main outcome (SDQ) is offered to participants who do not complete the full questionnaire passages at each measurement point. In cases where participants have not completed the pre-intervention questionnaire before the first group session, time is set apart for the family to complete the main outcome on site.

Newsletters from the project are published twice yearly on the study website (www.sibs.no) to keep participants informed about the study. All analyses will be done with treatment completers as well as the intent-to-treat sample.

See the “Recruitment {15}” section for further details regarding the retention and follow-up plans.

### Data management {19}

All questionnaire data will be collected electronically and transferred to TSD (Services for Sensitive Data), alongside video and audiotape data. TSD is part of the research infrastructure at the Department of Psychology. File backups are automatically generated every 14 days. Only anonymized data will be shared with collaborators. We have permission to store de-identified data until 2037.

### Confidentiality {27}

All data are stored in TSD, which is an enclosed electronic storage system at the University of Oslo. All access to this database is strictly regulated. Only de-identified data will be shared between collaborators.

### Plans for collection, laboratory evaluation, and storage of biological specimens for genetic or molecular analysis in this trial/future use {33}

This is not applicable, as no biological data will be collected.

### Statistical methods

#### Statistical methods for primary and secondary outcomes {20a}

The main effect is measured as the difference between the intervention group and the waitlist group in SDQ scores from pre-intervention to 3 months, reported by siblings, parents, and teachers. This is investigated using mixed ANOVA, and effect sizes will be reported as Cohen’s *d*. To examine the effects beyond waitlist for the complete sample, we apply growth curve modeling, which enables us to investigate both mean changes at a group level over time and individual growth trajectories, with the inclusion of individuals with partial/missing data [26]. A series of linear mixed models are fitted. A model with only fixed and random intercepts serves as the null model, to which subsequent unconditional models of time will be compared. Finally, group membership and control variables are included in a set of conditional models, through which the longitudinal effect of the intervention is investigated. Growth curve models are fitted separately for the intent-to-treat sample and per-protocol, excluding participants with concomitant sibling care during follow-up. Multilevel analyses controlling for nesting (group and site level) are conducted with MPlus. Other analyses are conducted using updated versions of IBM SPSS and R.

#### Interim analyses {21b}

We will not conduct interim analyses on outcomes. We will publish pre-intervention descriptive data. Thus, any decision to terminate the trial will be based on adverse events reported from the sites, and not be data-driven. The PI will make the decision to terminate the trial if required.

#### Methods for additional analyses (e.g., subgroup analyses) {20b}

We will analyze outcomes separately for different ND subgroups depending on sufficient *N*. We will also compare the results depending on the service tier the participants are recruited from (i.e., municipal or specialist health services).

#### Methods in analysis to handle protocol non-adherence and any statistical methods to handle missing data {20c}

Manual adherence is included as a predictor of outcomes. We report outcomes from all groups combined, but also examine if outcomes are different if groups with poor manual adherence are removed from the analyses. Our main strategy for handling missing data at the item level is through the default setting of the electronic questionnaires we use—these do not allow missing items. To handle missing data at the participant level, we apply growth curve modeling, which allows the inclusion of individuals with partial/missing data [26].

#### Plans to give access to the full protocol, participant-level data, and statistical code {31c}

The full protocol and the SIBS manual are available upon request. We will not share participant-level data, but de-identified group-level data and stat codes are available upon request.

### Oversight and monitoring

#### Composition of the coordinating center and trial steering committee {5d}

**Fig. 3:**
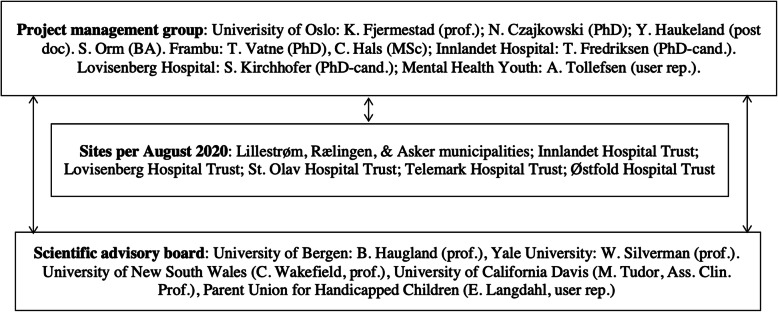
SIBS research group organization. rep, representative

See Fig. 3 for the overview of the multidisciplinary SIBS-RCT consortium. In the project management group, Czajkowski (PhD), expert in multilevel longitudinal analyses, is responsible for the statistics. Vatne (PhD), child psychologist with > 15 years of clinical experience and the first author of the SIBS manual, is responsible for the group leader training/supervision. Hals (MSc), journalist, is responsible for the e-learning/media communication. Fredriksen and Kirchhofer (both PhD candidates/clinical psychologists) and Haukeland (post-doc) are responsible for the data collection. Orm, research coordinator, coordinates the sites and manages the electronical data. Tollefsen (Mental Health Youth), user representative, is responsible for the user communication. The management group meets monthly and is supported by a scientific advisory board. This board will contribute to publications and be consulted in encountered challenges and comprises the following: Haugland (PhD, University of Bergen), expert in children who are next of kin. Silverman (prof.), expert in child mental health interventions, is responsible for the scientific publication progress and addressing the clinical research design issues. The Sydney team (Wakefield) are experts in family interventions for childhood cancer, including siblings. Tudor (UC Davis) is an expert in ND research, including siblings. Langdahl (Parent Union for Handicapped Children) is a user representative in the scientific advisory board.

Krister Fjermestad, professor, Department of Psychology, University of Oslo, is the principal investigator. He is a child psychologist with > 15 years of clinical experience alongside his research career. He has managed several ND-related research projects, including interventions. He is a co-author of the SIBS manual and was the group leader in our open trial.

### User involvement

A preliminary version of the SIBS intervention was piloted with 58 families in 2013 and adapted based on participant feedback (25). The adapted SIBS intervention was then delivered to 107 families in our open trial in collaboration with the national user unions for autism, Down syndrome, cerebral palsy, and congenital heart disease [11]. We made further adaptations (e.g., longer breaks between sessions; removing questionnaires) based on feedback from these unions and participants for the current SIBS-RCT. Board members from Mental Health Youth (Tollefsen) and the Parent Union for Handicapped Children (Langdahl) are represented in SIBS-RCT.

### Composition of the data monitoring committee, its role, and reporting structure {21a}

The project management acts as the data monitoring committee, but de-identified data will be made available upon request. There are no competing interests or sponsor conflicts.

### Adverse event reporting and harms {22}

Any adverse event will be handled in collaboration with the participating sites. All sites are prepared to offer additional counseling to participants who may need it. There were no adverse events during our open trial.

### Frequency and plans for auditing trial conduct {23}

No auditing is planned beyond regular project group meetings and regular contact with all sites.

### Plans for communicating important protocol amendments to relevant parties (e.g., trial participants, ethical committees) {25}

Any modifications will be reported to the ethical board and ClinicalTrials.gov if required. Newsletters from the trial will be sent to all participants twice yearly, in which potential changes will also be communicated.

### Dissemination plans {31a}

Communication aims include increased public awareness of sibling challenges; information about background, progress, and results of SIBS-RCT; and influencing policymakers to implement the SIBS intervention. The aims will be reached through publications of the following: online articles distributed via patient-user organizations, clinics, and political organizations; press releases/newspaper letters; articles in user unions’ member magazines; 6-monthly newsletters on the study website; a strategic document on siblings’ needs; > 10 scientific open access papers; > 10 presentations at research conferences; > 15 master student theses; and a summarizing user conference (2022). Our study website (www.sibs.no) includes links to publications and media coverage. We will publish positive, negative, and/or null findings. The project management group coordinates the publication plan, with authorships following Vancouver guidelines. The PhD candidates/post-doc have priority as the first authors. Conflicts will be handled by the project management with advice from the advisory board if required.

## Discussion

SIBS-RCT started participant recruitment and inclusion in September 2019. Recruitment is going according to plan, but many participants need several reminders before completing the online questionnaires. We believe this reflects the strain families of children with ND live under. We developed shorter versions of the questionnaire package to ensure we get as much data as possible on the main outcome variable, the SDQ.

SIBS-RCT corresponds to a societal need, in that Norwegian legislation requires health personnel to address the needs of siblings. Yet, ND and its impact on families represent a global challenge. We have tried the SIBS intervention with 54 families in Cambodia, with promising results on mental health, but not on family communication (submitted). Work is underway to adapt the manual further to the Cambodian culture. The team behind this proposal is interested in further international collaborations, and all materials are translated into English. For requests about implementation, contact the first or last author.

## Trial status

Inclusion started in September 2019. We aim to enroll 288 participants (288 siblings + 288 parents) by the end of 2021. The end of data collection including the 12-month follow-up will therefore be the end of 2023. Protocol version 1, September 2019.

## Abbreviations

BDI: Beck Depression Inventory
CAS-CBT: Competence and Adherence Scale for Cognitive Behavioral Therapy
KINDL: KINDL Fragebogen (German-named quality of life measure)
NAS: Negative Adjustment Scale
ND: Neurodevelopmental disorders
PCCS: Parent-Child Communication Scale
RCT: Randomized controlled trial
SCL-90: Symptom Checklist
SDQ: Strengths and Difficulties Questionnaire
SIBS-RCT: Group intervention for siblings and parents of children with chronic disorders – a randomized controlled trial
